# Large Retroperitoneal Liposarcoma Diagnosed upon Radiological Evaluation of Mild Right-Sided Inguinal Hernia

**DOI:** 10.1155/2013/187957

**Published:** 2013-12-03

**Authors:** Sophia K. McKinley, Nicolas Abreu, Eva Patalas, Arthur Chang

**Affiliations:** ^1^Harvard Medical School, Peabody Society, 260 Longwood Avenue, TMEC 253, Boston, MA 02115, USA; ^2^Department of Pediatrics, New York University School of Medicine, 550 First Avenue, New York, NY 10016, USA; ^3^Department of Pathology, Cambridge Health Alliance and Harvard Medical School, 1493 Cambridge Street, Cambridge, MA 02139, USA; ^4^Department of Radiology, Cambridge Health Alliance and Harvard Medical School, 1493 Cambridge Street, Cambridge, MA 02139, USA

## Abstract

While inguinal hernia is common in the primary care office, the differential diagnosis is extensive and includes infectious, inflammatory and neoplastic processes. Varicocele is another frequent, generally benign condition which occasionally reflects serious disease entities. Left-sided or bilateral varicoceles account for the overwhelming majority of varicoceles because the left gonadal vein drains into the left renal vein in contrast to the right gonadal vein, which drains directly into the inferior vena cava, thus making left-sided or bilateral venous congestion more likely. Presence of an uncommon unilateral right-sided varicocele thus warrants further radiological workup, in particular CT abdomen and pelvis, to evaluate for retroperitoneal pathology. We describe a case in which appropriate use of a variety of imaging modalities including testicular ultrasound and CT led to an important diagnosis of a large, well-differentiated liposarcoma in the right retroperitoneum of a patient with a right-sided groin mass.

## 1. Introduction

Depending on the source, liposarcoma is described as either the most common or second most common type of soft tissue sarcoma (STS) in adults comprising 24% of extremity STS and 45% of retroperitoneal STS [1, 2]. There is male predominance of cases ranging from a slight increase in incidence to a twofold incidence in men [3–5]. Additionally, incidence of liposarcoma increases with age with most cases presenting between 50 and 60 years of age. The etiology in most cases is unclear, and liposarcoma is not generally believed to arise from benign lipomatous tumors. However, an increasing number of studies are elucidating cytogenetic abnormalities associated with the different subtypes of liposarcomas [5, 6].

Liposarcomas can develop in any location in the body. The most common sites are the thigh and retroperitoneum. In the extremity, the tumor may present as a soft, painless mass which enlarges at any number of speeds ranging from slowly across years to rapidly across months. Retroperitoneal liposarcoma most often presents as an asymptomatic abdominal mass, though infrequently patients will present with symptoms caused by the effect of the growing mass on adjacent structures (incomplete obstruction, gastrointestinal bleeding, and pain) [5].

The World Health Organization categorizes liposarcoma into five distinct histologic subtypes: well differentiated, dedifferentiated, myxoid, pleomorphic, and mixed-type. CT and MR imaging findings may provide clues about the particular histology of a lesion suggestive of liposarcoma [1, 5, 7, 8]. The histologic subtype is important in determining a patient's prognosis [3–6].

The purpose of this case report is to describe how appropriate radiological workup of a patient who presented with a mild right-sided groin mass led to the diagnosis of a large, retroperitoneal well-differentiated liposarcoma which extended through the right inguinal canal.

## 2. Case Report

A 63-year-old gentleman was found by his primary care physician to have a new right inguinal canal impulse bulge upon presentation for an unrelated symptom. The patient was referred to a general surgeon, to whom he reported a one year history of an asymptomatic groin mass and possible urinary changes. On physical examination, the abdomen was soft, slightly obese, nontender, and nondistended. There was mild right testicular tenderness with a right inguinal canal impulse bulge. The left testicle was normal and there was no left inguinal canal impulse bulge.

**Figure 1:**
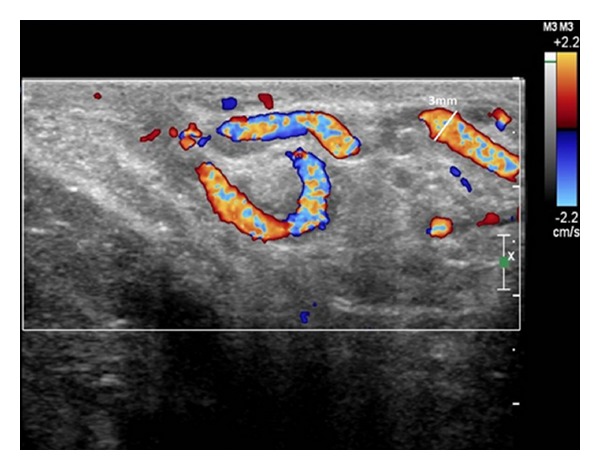
Right testicle doppler ultrasound, transverse superior view, showing right-sided varicocele with mild dilatation (3 mm) of vessels of the pampiniform plexus. There was no corresponding dilatation of vessels of the left pampiniform plexus.

Ultrasound ordered to evaluate hernia contents and rule out testicular pathology demonstrated a mild, unilateral right-sided varicocele measuring 3 mm (Figure 1). Otherwise, the exam was unremarkable: there were no focal lesions of either the right or left testicle and there was no definite bowel-containing hernia visualized on examination of the right scrotum.

**Figure 2:**
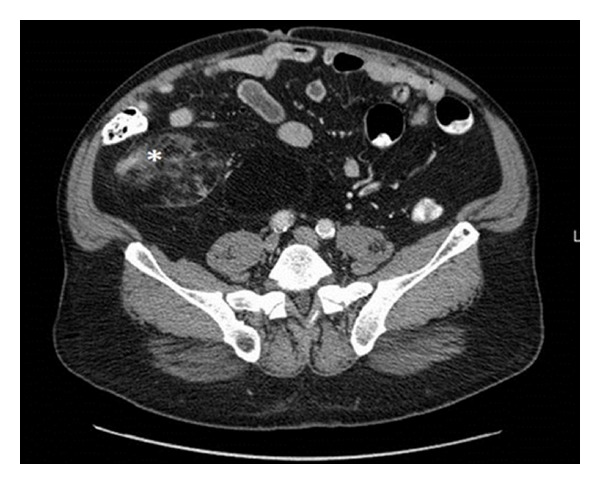
CT abdomen and pelvis with IV contrast, transverse image, displaying a large fatty lesion with associated soft tissue component (starred) in the right peritoneum anterior to the iliopsoas muscle. There is displacement of the bowel loops anteriorly and to the left.

**Figure 3:**
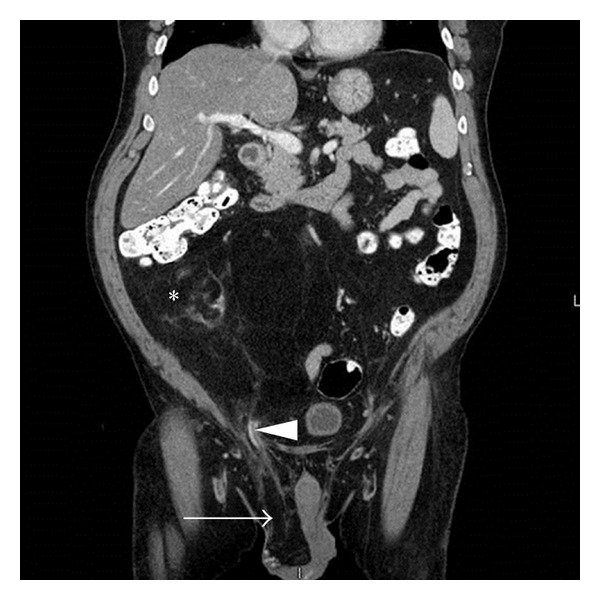
CT abdomen and pelvis with IV contrast, coronal image, demonstrating a large fatty lesion with associated soft tissue component in the right peritoneum extending into the right lower quadrant along the right paracolic gutter measuring 10.3 × 7.4 cm and 18.1 cm in superior to inferior direction. The lipomatous lesion extends into the inguinal canal (white arrowhead) resulting in right-sided inguinal hernia (white arrow). There is an area of ill-defined soft tissue component (starred). Bowel loops are displaced to the left.

A CT abdomen/pelvis with intravenous contrast was performed in order to rule out a mass in the right retroperitoneum that could have been compressing the right gonadal vein and causing venous congestion. This CT demonstrated a 10.3 × 7.4 × 18.1 cm predominantly fat density lesion with small internal focal areas of soft tissue density in the right retroperitoneum extending into the right lower quadrant along the right paracolic gutter and anterior to the iliopsoas muscle (Figures 2 and 3). The retroperitoneal location and presence of soft tissue components made liposarcoma much more likely than a benign lipoma [1].

The white arrow on Figure 3 highlights the right-sided inguinal hernia contents, which have the same homogenous hypointensity as the large fatty lesion in the retroperitoneum. The liposarcoma had likely extended through the inguinal rings resulting in indirect inguinal hernia appreciated on physical exam. Indicated by the white arrowhead in Figure 3, a section of the right gonadal vein courses through the deep inguinal ring where it was likely compressed by the liposarcoma, causing the patient's right-sided varicocele. There is also a nonlipomatous nodular focus of intermediate signal density seen starred in Figures 2 and 3 consistent with soft tissue elements. Additionally, the retroperitoneal tumor was exerting mass effect with leftward displacement of bladder and anterolateral displacement of bowel.

The patient's metastatic workup (chest CT with IV contrast) was negative and he underwent tumor resection. Surgical exploration demonstrated an obvious large, palpable, lobulated mass encapsulated within regular adipose tissue of the right retroperitoneum. The mass was removed with wide margins. Frozen section of the 17 × 17 × 8 cm specimen demonstrated adipose tissue with scattered chronic inflammation and rare histiocytes, though low-grade liposarcoma was not ruled out. The patient's postoperative course was unremarkable and he was discharged from the hospital on postoperative day 7.

**Figure 4:**
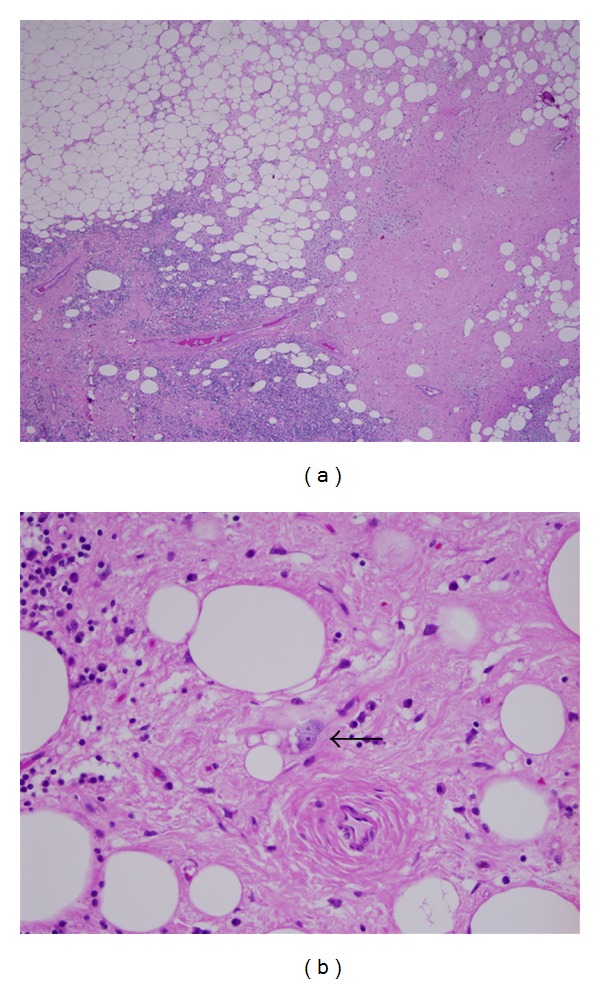
Photomicrograph of pathology of lipomatous retroperitoneal mass. Microscopic pathology. This is a composite photomicrograph which demonstrates representative findings from the initial surgical specimen in this case. (a) Low power representative field of the patient's surgical specimen which demonstrates adipocytes, sclerosis, and inflammation (hematoxylin and eosin stained section, 40x magnification). (b) Lipoblasts are indicated by the black arrow and while being a common feature of liposarcomas are not necessary for diagnosis of liposarcoma (hematoxylin and eosin stained section, 400x magnification).

Pathology confirmed the diagnosis of well-differentiated liposarcoma (Figure 4). The tumor was histologic grade 1 with a mitotic rate of 1/20 high-power fields in most cellular areas. No necrosis or lymphovascular invasion was identified. The superomedial, lateral, and inferomedial margins were positive on microscopy. The pathologic stage was T2bNxM0 and clinical stage 1 b, which is based on a deep tumor of size greater than 5 cm. Immunohistochemical stains performed on formalin fixed and paraffin embedded tissue showed that the highly atypical cells in the area of well-differentiated liposarcoma, inflammatory type, were negative for lymphoid marker CD45. Cytogenetic studies were attempted; however, the cells from the tumor specimen failed to proliferate in culture.

Due to positive microscopic margins, the patient proceeded to resection of residual disease including right orchiectomy, omental flap, and appendectomy at an outside, regional sarcoma center six months after the initial surgery. One microscopically positive margin persisted. The patient did not undergo any radiation or chemotherapy as part of his treatment.

Now two and a half years after his initial diagnosis, this gentleman continues to be monitored for local and distant recurrence of disease with biannual abdominal/pelvic CTs and annual chest X-rays.

## 3. Discussion

Patients are frequently seen by primary care physicians and general surgeons for the evaluation of a groin mass. Inguinal hernia is a common cause of a bulge in the groin and the differential diagnosis for hernia sac contents extends beyond fat and bowel, including intraperitoneal hemorrhage from ruptured abdominal aortic aneurysm or splenic rupture, metastatic deposits, abdominal tuberculosis, ascites, appendicitis, appendicular abscess, endometriosis, and even uterus in pseudohermaphrodite [9–12]. While a variety of imaging modalities are available, ultrasound is the first choice in the evaluation of a groin mass due to cost, safety, availability, and high sensitivity and specificity.

This patient's groin mass was initially evaluated by testicular ultrasound, which demonstrated no testicular lesions or definite bowel-containing hernias. However, there was a mild unilateral right-sided varicocele. Unilateral right-sided varicoceles constitute only 7% of all varicoceles. Varicoceles are most frequently unilateral left-sided (68%) or bilateral (25%) due to the difference in venous drainage of the right and left testicles [13–16]. In particular, the left gonadal vein drains first into the left renal vein, whereas the right gonadal vein drains directly into the inferior vena cava. Therefore, unilateral left-sided varicocele is not worrisome because it is most likely caused by congestion due to drainage into a higher resistance vessel. Unilateral right-sided varicocele can also indicate a benign process such as incompetent right gonadal vein valves or anomalous insertion of the right gonadal vein into the right renal vein but can portend a retroperitoneal neoplastic process resulting in venous compression [14]. Imaging by CT abdomen/pelvis is recommended to rule out a retroperitoneal mass because it allows for soft tissue resolution and well defines the anatomic location of soft tissue tumors relative to gonadal veins. In addition to being cheaper and more available than MRI, CT is less sensitive to motion artifact.

The patient we describe appropriately underwent CT abdomen/pelvis to rule out right retroperitoneal pathology and was found to have a large right retroperitoneal lipomatous mass, most likely liposarcoma, which extended into the scrotum and could account for both the physical exam finding of right inguinal hernia and the unilateral right-sided varicocele. Pathology ultimately confirmed the diagnosis of well-differentiated liposarcoma. In retrospect, the liposarcoma was not detected in the scrotum by the initial ultrasound as the mass was fatty and indistinguishable from normal adipose tissue. It was also likely nonmobile, which would make it difficult to detect on valsalva as opposed to mobile, fat-containing inguinal hernia.

Well-differentiated liposarcoma accounts for approximately 50% of liposarcomas, with the most common site being the lower extremity (50%) followed by the retroperitoneum (20%) [1, 5, 7]. Histologically, well-differentiated liposarcoma is very similar to normal adipose tissue and is composed primarily of mature adipocytes [1, 13, 15]. However, these adipocytes may vary considerably in size and have nuclear atypia. Lipoblasts may be a feature of well-differentiated liposarcoma but are not required for diagnosis. Well-differentiated liposarcoma is subcategorized into lipoma-like, sclerosing, inflammatory, or spindle cell depending on additional features which are present or absent. Considered a lower grade tumor than dedifferentiated, myxoid, round cell, and the pleomorphic types of liposarcoma, well-differentiated liposarcoma has a high rate of local recurrence but does not have metastatic potential [5, 7].

On CT and MR, well-differentiated liposarcoma appears as a predominantly adipose soft tissue mass with nonlipomatous components [1, 7]. These nonlipomatous features include septa (often >2 mm) and/or small (<2 cm) foci of nodular or globular nonadipose tissue. Additionally, calcifications may be present within the lesion. Large size and nonlipomatous elements such as thick septa distinguish well-differentiated liposarcoma from lipoma on CT and MR [1, 7]. Gadolinium contrast enhancement may also help clarify whether a lesion is lipoma or liposarcoma: the majority of lipomas demonstrate no contrast enhancement whereas the majority of liposarcomas demonstrate moderate to marked enhancement of septa [8]. On ultrasound, liposarcoma appears as a well-defined, multilobulated soft tissue mass. Hyperechoic foci suggestive of fat may indicate that the mass is lipomatous in nature, but ultrasonography is a poor technique at distinguishing liposarcoma from lipoma [1]. The present patient's imaging findings are consistent with well-differentiated liposarcoma, a large, lipomatous mass with nonlipomatous components including septa and nodular/globular foci.

The large size of the nonlipomatous tissue foci suggested dedifferentiated liposarcoma. Because dedifferentiated liposarcoma arises within the context of well-differentiated liposarcoma, most of the radiological features are the same. However, nodules of nonlipomatous tissue >2 cm in size can indicate that the lesion is dedifferentiated liposarcoma, though this diagnosis must be confirmed histologically [1]. MR is better suited than CT for evaluating these nonadipose components due to its ability to better discriminate among soft tissues. Dedifferentiated liposarcoma has low to intermediate signal intensity on T1-weighted MR and higher signal intensity on T2-weighted MR imaging [1].

Clues about the histological subtype of liposarcoma are especially critical given that it is the most important prognostic factor. Outcomes vary widely depending on the liposarcoma subtype: well-differentiated liposarcoma has the best prognosis with five-year survival rates of 90% or higher whereas pleomorphic liposarcoma has five-year survival rates reported to be as low as 30% [3–6]. Patients with liposarcoma of the extremity have improved survival compared to patients with retroperitoneal liposarcoma [5]. Risk of recurrence also depends on tumor histology and location. Retroperitoneal well-differentiated liposarcoma has a recurrence rate of over 90% versus 43% for an extremity lesion [1]. Dedifferentiated liposarcoma in the retroperitoneum has a nearly 100% recurrent rate. Contributing to the high recurrence rate of tumors of the retroperitoneum is the difficulty in attaining negative surgical margins.

Complete resection of the tumor with wide margins is the primary treatment of liposarcoma [1, 2, 5]. In the extremities, the goal is to excise the tumor and a cuff of normal tissue. For retroperitoneal liposarcoma, achieving a negative margin may require en bloc resection of involved organs such as the kidney [5]. The use of chemotherapy or radiation therapy in the treatment of liposarcoma is dependent on the tumor grade and location [1, 5]. For retroperitoneal liposarcoma, use of radiation therapy to improve local control has often failed to demonstrate any survival benefit. However, radiation therapy has been shown to provide benefit for extremity liposarcoma of large size or high histologic grade. Adjuvant chemotherapy has been found to have a survival benefit in myxoid and pleomorphic liposarcoma, as these are high grade tumors with high metastatic potential [1, 5].

The differential diagnosis of lipomatous tumors includes lipoma, the five types of liposarcoma, hibernoma, and lipoblastoma [5, 7]. On CT or MR, myxoid liposarcoma appears as a well-defined, multilobulated, large intramuscular lesion with a characteristic lacy/linear fat pattern. Additionally, myxoid liposarcoma may demonstrate high signal on T2-weighted MRI that resembles a cyst [1]. Pleomorphic liposarcoma is not predominantly lipomatous. Instead it appears as nonspecific soft tissue with foci of fat, necrosis, and/or hemorrhage. This variety of tissue elements leads to a heterogeneous appearance on CT and MR. Mixed-type liposarcoma has a highly variable appearance on imaging, as it demonstrates features of the four other types of liposarcoma and its imaging findings will depend on the tumor's particular histologic composition. Hibernoma is a peculiar tumor of brown fat that occurs most commonly in the thigh of adults and is cured with complete excision [17]. Lipoblastoma is a benign tumor that develops from immature adipocytes in young children [18].

In summary, we report a case of a large, well-differentiated liposarcoma in the right retroperitoneum that was diagnosed as a result of thorough follow-up of incidental right-sided inguinal hernia, including imaging studies. The hernia was identified by the patient's primary care physician during evaluation for another complaint. This case demonstrates (1) the importance of thorough physical examination and (2) the need to avoid premature closure in diagnosis of groin masses. Not all groin masses are simple hernias, and hernia cases have the potential to reflect distant disease processes. The rarity of a right-sided varicocele reflects the fact that the right gonadal vein drains directly into the inferior vena cava and is therefore much less likely to manifest venous congestion in the absence of left-sided congestion. Unilateral right-sided varicocele warrants CT follow-up to rule out retroperitoneal pathology causing compression of the right gonadal vein. In this case, CT also provided valuable insight into the histology of the discovered retroperitoneal lesion.
